# Enterotoxigenic *Bacteroides fragilis* activates IL-8 expression through Stat3 in colorectal cancer cells

**DOI:** 10.1186/s13099-022-00489-x

**Published:** 2022-04-25

**Authors:** Rachel V. Purcell, Jessica Permain, Jacqueline I. Keenan

**Affiliations:** Department of Surgery, University of Otago, Christchurch, New Zealand

**Keywords:** Enterotoxigenic *Bacteroides fragilis*, Toxin, Interleukin-8, IL-8, Stat3, E-cadherin, Colorectal cancer, Inflammation, HT29, HCT116, β-catenin

## Abstract

**Background:**

Enterotoxigenic *Bacteroides fragilis* (ETBF) has been implicated in colorectal carcinogenesis through the actions of its toxin, *B. fragilis* toxin (BFT). Studies on colorectal cell lines have shown that treatment with BFT causes disruption of E-cadherin leading to increased expression of the pro-inflammatory cytokine, IL-8. Stat3 activation has also been associated with ETBF-related colitis and tumour development. However, a link between E-cadherin, IL-8 and Stat3 has not been investigated in the context of ETBF infection.

**Results:**

We found that co-culture of HT-29 and HCT116 colorectal cell lines with ETBF, had a similar effect on activation of IL8 gene and protein expression as treatment with purified BFT. Inhibition of Stat3 resulted in a decrease in IL-8 gene and protein expression in response to ETBF in both cell lines. A reduction in E-cadherin expression in response to ETBF treatment was not restored by blocking Stat3.

**Conclusion:**

We found that treatment of colorectal cancer cell lines with live cultures of ETBF had the equivalent effect on IL-8 expression as the use of purified toxin, and this may be a more representative model of ETBF-mediated colorectal carcinogenesis. IL-8 gene and protein expression was mediated through Stat3 in HT-29 and HCT116 cells, whereas disruption of E-cadherin appeared to be independent of Stat3 signalling.

**Supplementary Information:**

The online version contains supplementary material available at 10.1186/s13099-022-00489-x.

## Background

Enterotoxigenic *Bacteroides fragilis* (ETBF) has been implicated in colorectal carcinogenesis through the actions of its toxin, *B. fragilis* toxin (BFT), on multiple molecular processes [1]. Studies using both in vitro cell line and in vivo models have identified disruption of E-cadherin, an important regulator of epithelial-mesenchymal transition (EMT), and initiation of pro-inflammatory cascades to be involved in ETBF-mediated carcinogenesis [2–4]. Several in vitro studies of colonic epithelial cells (CECs), predominantly using HT-29 CRC cell lines, have demonstrated an increase in interleukin (IL)-8 expression in response to purified BFT [4, 5].

IL-8 is a potent inflammatory chemokine, and its secretion by CECs leads to downstream activation of numerous signalling pathways involved in cell survival, angiogenesis and cell migration/invasion. BFT has been shown to stimulate the secretion of IL-8 through E-cadherin disruption [5], which releases bound β-catenin and allows nuclear translocation and subsequent TCF-dependant transcription of IL-8 [6]; treatment of CECs with BFT has been reported to increase β-catenin pathway activation [7]. β-catenin signalling is believed to have a role in modulating acute inflammation [8, 9], which may, in turn, facilitate chronic inflammation, hypothesised to play a role in carcinogenesis. IL-8 signalling has also been shown to activate Stat3 [10], which regulates various cell functions, including acute inflammation.

Stat3 can act in two ways: by transducing extracellular signals, often from cytokine receptors, in a feed-forward loop, and also by regulating the expression of a large number of genes by acting as a transcription factor. When Stat3 is constitutively activated, it acts as an oncogene, promoting chronic inflammation and tumourigenesis [11], and recent evidence suggests that Stat3 may be involved in skewing the host response towards tolerance at the cost of immunity in Helicobacter pylori [12] and ETBF [13] infections. Stat3 activation has also been associated with ETBF-associated colitis [14] and colonic tumour development in mice [13]. The interplay between IL-8 and Stat3 [15, 16], and E-cadherin and Stat3 [17, 18] has been described in other types of cancer, but the potential interaction linking these molecular pathways has not been interrogated in the context of ETBF-mediated colorectal tumourigenesis.

The inflammatory effect of ETBF-related tumourigenesis has been described in detail, from studies carried out in mouse models, particularly in APC-min mice [2, 3, 19]. However, cell line models of ETBF-related CRC have relied solely on the use of purified BFT to mimic the effect of the bacteria. This approach may not adequately account for modifying factors present in whole bacterial preparation, such as cell-wall components, or account for the fact that ETBF does not always secrete toxin in vivo.

Here, we investigate the effect of ETBF cultures compared to purified BFT on the activation of molecular pathways involved in ETBF-related pathogenesis. This study also elucidates the interplay between IL-8, Stat3 and E-cadherin in response to ETBF in colorectal cell lines.

## Methods

### Bacterial cell culture

Enterotoxigenic *B. fragilis* (ETBF) strain 86-5443-2-2 was cultured in brain–heart-infusion broth enriched with 0.2% heme iron (Sigma-Aldrich, St Louis, MO, US). Bacterial concentration was measured by spectral absorbance. In brief, 1 ml of bacterial broth was centrifuged at 10,000 rpm for 5 min and the supernatant discarded, and the bacterial pellet resuspended in 1 ml of PBS. Following a further centrifugation, the supernatant was removed, and the bacteria resuspended in 1 ml of PBS. This bacterial suspension was further diluted 1:10 and 1:5 in PBS and the absorbance measured in 600 nm using a SkanIt plate reader (Thermo Fisher, Waltham, MA, US). An absorbance reading at 600 nm of 0.1 corresponds to 1 × 10^8^ bacteria.

### Colorectal cancer cell culture

HT-29 and HCT116 cells (American Type Culture Collection (ATCC), Manassas, VA, US) were cultured in McCoy’s medium (Thermo Fisher), containing 10% foetal bovine serum and 1% penicillin/streptomycin and grown in 5% CO_2_. For all experiments, cells were grown in 24-well plates and seeded at a density of 5 × 10^4^ cells/well. These cell lines have an approximate doubling time of 24 h, giving 2 × 10^5^ cells/well after 48 h incubation. Cells were harvested, washed in PBS and stored in RNAlater (Qiagen, Hilden, Germany) at − 20 °C for subsequent RNA extraction and gene-expression analysis. Cell supernatants were removed directly from culture wells and stored at − 20 °C for ELISA analysis, while SDS buffer (60 mM Tris pH 4.8, 2% SDS, 20% glycerol, 0.1 M DTT, 1 × protease inhibitor; Thermo Fisher) was added to 80% confluent cells and directly harvested for subsequent Western Blot analysis.

### IL-8 protein detection

An enzyme-linked immunosorbent assay (ELISA) to detect IL-8 protein was performed using Human IL-8/CXCL8 DuoSet ELISA kit (R + D Systems, Minneapolis, MN, US) according to the manufacturer’s instructions. Briefly, a 96-well microplate was coated with 100 μL per well of the diluted capture antibody, overnight at room temperature, before aspirating and washing. The microplate was blocked by adding 300 μL of block buffer to each well, and incubated at room temperature for 1 h, followed by aspiration and washing. 100 μL of sample or standards was added to each well and incubated for 2 h at room temperature, followed by aspiration and washing. Detection Antibody (100 μL) was then added to each well and incubated for 2 h at room temperature, followed by aspiration and washing. Streptavidin-HRP was added (100 μL) and incubated for 20 min in the dark, before washing. Substrate solution was then added to each well for 20 min in the dark, followed by 50 μL of Stop Solution. The optical density of each well was read immediately, using a SkanIt microplate reader set to 450 nm.

### Quantitative gene expression analysis

RNA was extracted from cultured cells using RNeasy Mini Kit (Qiagen), as per the manufacturer’s instructions. RNA was reverse transcribed using qScript cDNA SuperMix (Quantabio, Beverley, MA, US), using 2ul of sample and 18ul of master mix. Quantitative real-time polymerase chain reaction (qPCR) was carried out to quantify levels *CXCL8* using the LightCycler®480 thermocycler (Roche Diagnostics, Indianapolis, IN, USA) using SYBR-green chemistry. Each 10 µl reaction consisted of 25–35 ng of genomic DNA, 0.5 µM of each primer pair, 5 µl of SYBR Green Master Mix (Roche Diagnostics), and 1.5 µl of water. Thermal cycling conditions were as follows: 1 cycle of 95 °C for 5 min, followed by 60 cycles of 95 °C for 10 s, 58 °C for 10 s and 72 °C for 20 s. Melting curves were obtained by heating samples from 65 to 97 °C in increments of 0.11 °C/s with 5 acquisitions per °C. Each analysis was carried out in triplicate, with *HPRT* as a reference gene. Primer sequences are available in Additional file 1.

### Western blot analysis

Proteins from the cell lysates were separated with SDS-PAGE based on the Laemmli method, using Bio-Rad Mini-Protean system. In brief, samples were denatured for 5 min at 95 °C prior to SDS-PAGE analysis using pre-cast gradient gels (Bio-Rad, Hercules, CA, US) at 200 V for 40 min. The separated proteins were then transferred to 0.4 μm PVDF membrane (GE Healthcare, Chicago, IL, US) with Mini-Trans-Blot Cells in cold Western Blot transfer buffer (25 mM Tris, 192 mM glycine, pH 8.3, and 10% methanol) at 100 V for 90 min based on the size of the protein of interest. Membranes were blocked with non-fat milk powder (5%) in Tris buffered saline with 0.05% (v/v) Tween-20 (TBST) for 1 h at room temperature. The membranes were then incubated with primary antibodies diluted in non-fat milk overnight at 4 °C. Primary antibodies used were anti-E-cadherin mouse monoclonal (Abcam, Cambridge, UK; ab76055), anti-Stat3 mouse monoclonal (Abcam, ab119352), anti-β-catenin rabbit monoclonal (Abcam, ab32572) and anti-β-actin mouse monoclonal (Sigma-Aldrich, A5441).

The membrane was washed with TBST three times before incubation with the appropriate goat anti-mouse or goat anti-rabbit secondary antibody for 1 h at 1:10,000 dilution, followed by washing and chemiluminescent detection using ECL Plus detection reagent (GE Healthcare). The signal was visualised, and the expression of proteins quantified with the NineAlliance Q9 (UVITECH, Cambridge, UK). Proteins were normalized to the β-actin band on each lane.

### Statistical analyses

Two-way ANOVA followed by Dunnett’s multiple comparisons test was performed using GraphPad Prism version 8.0.0 for Windows (GraphPad Software, San Diego, California USA, www.graphpad.com). Findings were considered significant at a *P-*value of < 0.05.

## Results

### ETBF co-cultured with CRC cell lines induces IL-8 expression

**Fig. 1:**
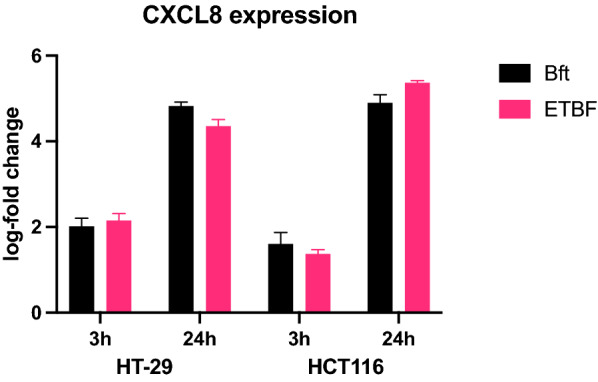
*CXCL8* expression in HT-29 and HCT116 cells, following treatment with *Bacteroides fragilis* toxin (BFT, 1 ug/ml) or enterotoxigenic *Bacteroides fragilis* (ETBF, MOI 100:1) for 3 or 24 h

HT-29 and HCT116 cells were incubated with either ETBF at a multiplicity of infection (MOI) of 100:1 or purified *B. fragilis* toxin (BFT; 1 ug/ml in McCoy’s media) or control (McCoy’s media). Cells were incubated for 3 or 24 h, supernatants removed, and cells harvested. Relative expression of *CXCL8* (the IL-8 gene) was measured by qPCR. After three hours both ETBF and BFT induced a twofold increase in *CXCL8* expression compared to control in HT29 cells, which increased to more than fourfold increase after 24 h. In HCT116 cells a 1.5-fold and fivefold increase in *CXCL8* expression was detected at 3 and 24 h, respectively (Fig. 1). All subsequent experiments were carried out using 24-h incubations. Levels of secreted IL-8 protein were measured in the supernatants of cell lines treated with ETBF or BFT for 24 h using an ELISA method. An approximately twofold increase in IL-8 protein was detected in both cell lines after ETBF or BFT treatment, compared to control.

### Induction of IL-8 expression is STAT3 dependent

**Fig. 2:**
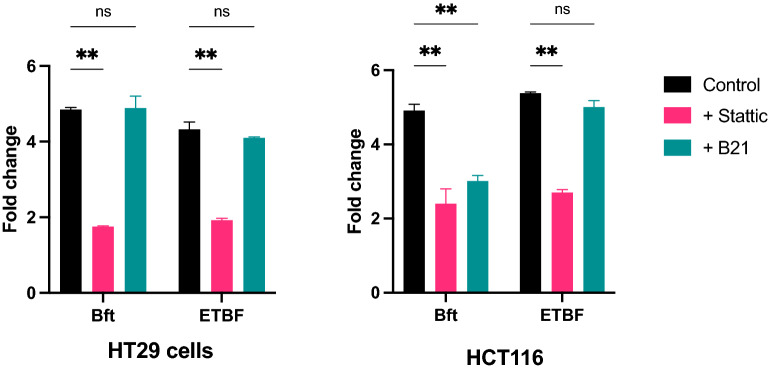
Changes in *CXCL8* expression in **A** HT29 and **B** HCT116 cells induced by *Bacteroides fragilis* toxin (Bft) or Enterotoxigenic *Bacteroides fragilis* (ETBF), following treatment with Stat3 antagonist (Stattic) or Beta-catenin antagonist (B21). ns, non-significant; **P-value < 0.005

In order to investigate the potential molecular pathways involved in the induction of IL-8 expression by ETBF/BFT, we pre-treated CRC cell lines with antagonists to Stat3 (Stattic) and β-catenin (B21) prior to incubation with ETBF or BFT. Both antagonists were optimised for use at 10 µM. HT-29 and HCT116 cells were pre-treated with antagonists ( ±) for 1 h, followed by washing in PBS and co-incubation with ETBF/BFT/control for 24 h as detailed above. Supernatants were collected and frozen for protein analysis and RNA extracted from harvested cells for gene-expression analysis. Pre-treatment with Stat3 antagonist significantly reduced expression of *CXCL8* in both cell lines, when cells were cultured with either ETBF or BFT. Blocking β-catenin activity did not lead to a comparable reduction in *CXCL8*, although a non-significant reduction was seen in HCT116 cells co-cultured with BFT (Fig. 2). Subsequent analyses were carried out using ETBF only and not BFT.

**Fig. 3:**
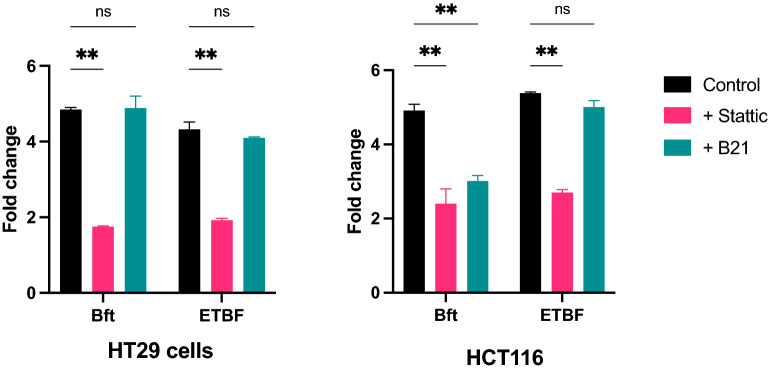
Changes in secreted IL-8 protein expression in **A** HT29 and **B** HCT116 cells induced by enterotoxigenic *Bacteroides fragilis* (ETBF), following treatment with Stat3 antagonist (Stattic) or β-catenin antagonist (B21). ns, non-significant; * *P*-value < 0.05; *** *P*-value < 0.0005

Similar to gene-expression analysis, protein expression of IL-8 was decreased in response to ETBF when cells were pretreated with Stat3 inhibitor. The reduction in IL-8 protein was less marked in HCT116 cells in response to BFT. Blocking β-catenin, using B21, did not have a significant impact on IL-8 protein expression (Fig. 3) (Additional file 2: Table S1).

### Treatment with ETBF leads to a reduction in E-cadherin expression

**Fig. 4:**
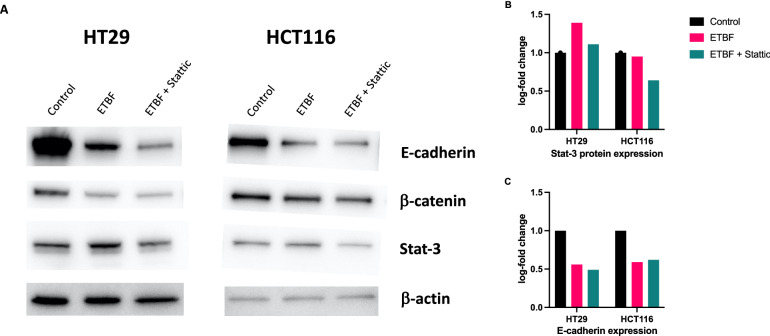
**A** Western Blot of HT29 (left) and HCT116 (right) colorectal cancer cell lines probed with antibodies to E-Cadherin, β-catenin and Stat3. β-actin was used as a loading control. Relative quantification of **B** Stat-3 and **C** E-cadherin expression derived from Western blot analysis in HT29 and HCT116 cells

Treatment of cell lines with ETBF resulted in a reduction of E-cadherin protein in both HT-29 and HCT116 cell lines (Fig. 4). Blocking Stat3 using a Stat3 antagonist did not restore E-cadherin expression, suggesting that the effect of ETBF on E-cadherin is not mediated through Stat3. Surprisingly, expression of β-catenin was slightly reduced in HT-29 cells in response to ETBF treatment, whereas this effect was not observed in HCT116 cells.

## Discussion

In the current study, we have investigated the effect of co-culturing with live strains of enterotoxigenic *Bacteroides fragilis* (ETBF) compared to purified toxin from the bacterium (BFT) in colorectal cancer (CRC) cell lines. ETBF has been implicated in CRC pathogenesis through the action of its toxin on E-cadherin and several inflammatory pathways, including upregulation of IL-8. Studies to date have predominantly used HT-29 and HCT116 CRC cell lines and treatment with purified BFT to investigate ETBF-related colorectal carcinogenesis [4, 5, 20, 21]. Recent studies, however, have suggested that *Bacteroides* species may have an anti-inflammatory effect on gut mucosa, through the action of cell-wall components, such as lipopolysaccharide [22], and using purified toxin in experimental systems may not account for the nuanced effect of the bacterium on pro- and anti-inflammatory pathways. Non-toxigenic *B. fragilis* can reduce ETBF-mediated colitis in a mouse model, demonstrating that anti-inflammatory components of *B. fragilis* may play a physiological role in mitigating toxin-induced inflammation by polysaccharide A-dependent [23] and independent mechanisms [24]. In addition, the bacterial toxin may not be expressed in established CRC, despite the presence of the bacteria in tumour samples (unpublished data). Using IL-8 induction as a measure of ETBF-mediated molecular changes, we treated HT-29 and HCT116 cell lines with purified BFT and ETBF at a multiplicity of infection of 100:1. We found that treatment with ETBF had a similar effect on gene (*CXCL8*) and protein expression of IL-8, compared to purified toxin, highlighting the utility of using a bacterial culture rather than purified toxin.

We next sought to elucidate the interplay between IL-8 and Stat3 in ETBF-related colorectal carcinogenesis. As a key component of the JAK/STAT pathway, Stat3 has been reported to be constitutively activated in several studies of CRC [25–27]. Disruption of Stat3 signalling leads to tumour-cell invasion through disruption of cell adhesion complexes, including E-cadherin, a crucial event of epithelial-mesenchymal transition in cancer [17, 28, 29]. ETBF has been shown to induce persistent colitis in mice, with increased Stat3 expression and mucosal permeability [13], independent of Stat3 activation in colonic epithelial cells in a study by Wick et al. [14].

IL-8 is upregulated in many cancers and has tumourigenic effects via angiogenesis, neutrophil recruitment and proliferation and migration of tumour cells [16, 30–32]. IL-8 also activates classic inflammatory signalling pathways, such as NF-κB [33], while its expression is induced by other inflammatory pathways, such as MAPK [34], suggesting that IL-8 may play a role in chronic inflammation-related cancer. BFT has previously been reported to induce expression of IL-8 gene and protein in CRC cell lines [4, 21] and this increased expression was shown to occur through disruption of E-cadherin junctions, although cleavage of E-cadherin is not required [5]. We saw a similar increase of both IL-8 gene and protein expression in response to treatment with both ETBF and BFT. In order to ascertain the role of Stat3 in ETBF-mediated IL-8 signalling, we used to Stat3 antagonist to block Stat3 signalling. This resulted in a significant decrease in gene and protein expression of IL-8 in both CRC cell lines, suggesting that IL-8 signalling in response to ETBF is dependent on Stat3. BFT has been shown to induce IL-8 through MAPK and NF-kB in HT29 cells [4]. Stat3-mediated gene expression and tumourigenesis can also be activated by MAPK and NF-kB [35, 36]. Our findings suggest that Stat3 may be a crucial mediator of MAPK and NF-kB signalling in response to ETBF that leads to downstream IL-8 expression that may, in a similar manner to IL-6 [37], promote a feed-forward loop in inflammation.

Loss of E-cadherin expression is a crucial step and fundamental feature of epithelial-to-mesenchymal transition (EMT) in cancer progression, and this disruption of E-cadherin has been demonstrated in response to BFT in numerous studies using CRC cell lines [20, 38, 39]. However, although E-cadherin disruption and subsequent invasion has been shown to be induced by Stat3 in CRC cell lines [25], this has not been demonstrated in the context of ETBF. Our findings also demonstrated that treatment with ETBF resulted in decreased expression of E-cadherin in HT-29 and HCT116 cells. However, blocking Stat3 using a Stat3 antagonist did not restore E-cadherin expression, suggesting that the effect of ETBF on E-cadherin is not mediated through Stat3.

In vitro investigations of ETBF/BFT in CRC have predominantly been carried out using the HT-29 cell line. Our findings of subtle differences in the expression of β-catenin in response to ETBF in HCT116 cells compared to HT-29 cells emphasises the limitations of extrapolating data from a single cell line. One important difference between these cell lines is that HT29 cells have a mutated *APC* gene whereas HCT116 retains wildtype *APC*, and this may impact β-catenin, through its direct interaction with APC in the nucleus. Differences in molecular mechanisms at play in the colonic mucosa and within different molecular subtypes of CRC may impact how an individual tumour responds to an assault by ETBF. Crosstalk between different cell types in the tumour microenvironment may also play an important role in cell signalling, as previously shown by the activation of immune-cell Stat3, independent of activation of epithelial cell STAT3 and subsequent increase in mucosal permeability, in a mouse model of ETBF response [14]. Future studies into the role of Stat3 in IL-8 signalling in response to ETBF should use co-cultures of CRC and immune cells or a mouse model to investigate crosstalk between different cell types.

## Conclusion

We found that treatment of CRC cell lines with live cultures of ETBF had the equivalent effect on IL-8 expression as the use of purified toxin, and this may be a more representative model of ETBF-mediated colorectal carcinogenesis. IL-8 gene and protein expression was mediated through Stat3 in HT-29 and HCT116 cells, whereas disruption of E-cadherin appeared to be independent of Stat3 signalling.

## Supplementary Information

**Additional file 1.** Primer sequences used in the study.

**Additional file 2. Table S1.** IL-8 protein expression values from multiple ELISA analyses.

## Data Availability

The dataset supporting the conclusions of this article is included within the article (and its Additional file).
