# Supplementary material for: Enterotoxigenic Bacteroides fragilis activates IL-8 expression through Stat3 in colorectal cancer cells
Source: Gut Pathog. 2022 Apr 25;14:16. doi: 10.1186/s13099-022-00489-x (PMC9036718; doi:10.1186/s13099-022-00489-x)
Supplement: Supplementary file 1 — Additional file 1. Primer sequences used in the study. [file 13099_2022_489_MOESM1_ESM.docx]

Additional file 1: primer sequences for PCR

*CXCL8*

Forward: 5′-ATGACTTCCAAGCTGGCCGTGGCT-3′

Reverse: 5′-TCTCAGCCCTCTTCAAAAACTTCT-3′.

*HPRT*

Forward: 5’-CAGTCCCAGCGTCGTGATTA -3’

Reverse: 5’-TGGCCTCCCATCTCCTTCAT-3’
