# Supplementary material for: Enterotoxigenic Bacteroides fragilis activates IL-8 expression through Stat3 in colorectal cancer cells
Source: Gut Pathog. 2022 Apr 25;14:16. doi: 10.1186/s13099-022-00489-x (PMC9036718; doi:10.1186/s13099-022-00489-x)
Supplement: Supplementary file 2 — Additional file 2. Table S1. IL-8 protein expression values from multiple ELISA analyses. [file 13099_2022_489_MOESM2_ESM.docx]

|  |  | **Experiment 1** | |  | **Experiment 2** | |  | **Experiment 3** | |
| --- | --- | --- | --- | --- | --- | --- | --- | --- | --- |
|  |  | **Average pg/ml** | **Fold Change** |  | **Average pg/ml** | **Fold Change** |  | **Average pg/ml** | **Fold Change** |
| **HT29** | **Control** | 2.56 | 1 |  | 35.91 | 1 |  | 10.37 | 1 |
|  | **ETBF** | 5.88 | 2.29 |  | 72.57 | 2.02 |  | 25.09 | 2.42 |
|  | **Stattic +ETBF** | 3.89 | 1.52 |  | 44.7 | 1.24 |  | 14 | 1.35 |
|  |  |  |  |  |  |  |  |  |  |
|  |  |  |  |  |  |  |  |  |  |
|  |  | **Average pg/ml** | **Fold Change** |  | **Average pg/ml** | **Fold Change** |  | **Average pg/ml** | **Fold Change** |
| **HCT116** | **Control** | 1.11 | 1 |  | 14.12 | 1 |  | 52.91 | 1 |
|  | **ETBF** | 1.15 | 1.04 |  | 16.67 | 1.18 |  | 62.31 | 1.18 |
|  | **Stattic +ETBF** | 0.88 | 0.88 |  | 15.23 | 1.08 |  | 58.52 | 1.11 |
